# Designing an early selection morphological traits index for reproductive efficiency in Pura Raza Española mares

**DOI:** 10.1093/jas/skad409

**Published:** 2023-12-20

**Authors:** Davinia I Perdomo-González, María J Sánchez-Guerrero, Ester Bartolomé, Rute Guedes dos Santos, Antonio Molina, Mercedes Valera

**Affiliations:** Departamento de Agronomía, Escuela Técnica Superior de Ingeniería Agromómica, Universidad de Sevilla, Sevilla 41013, Spain; Departamento de Agronomía, Escuela Técnica Superior de Ingeniería Agromómica, Universidad de Sevilla, Sevilla 41013, Spain; Departamento de Agronomía, Escuela Técnica Superior de Ingeniería Agromómica, Universidad de Sevilla, Sevilla 41013, Spain; Polytechnic Institute of Portalegre, Portalegre 7300-110, Portugal; Research Centre for Endogenous Resource Valorization (VALORIZA), Portalegre 7300-555, Portugal; Departamento de Genética, Universidad de Córdoba, Córdoba 14014, Spain; Departamento de Agronomía, Escuela Técnica Superior de Ingeniería Agromómica, Universidad de Sevilla, Sevilla 41013, Spain

**Keywords:** biometric traits, equine, fertility traits, genetics parameters, selection index

## Abstract

The low rate of reproductive efficiency in horses may be linked to the equine industry’s practice of maintaining breeding stock that stands out for its athletic or morphological performance but exhibits poor reproductive efficiency. In this study, the age at first foaling, age at last foaling, interval between first and second foaling, average interval between foaling, foaling number and reproductive efficiency, and their relationship with the morphological records in 19,758 Pura Raza Española (**PRE**) mares were analyzed. After a partial least squares analysis height at withers, lateral hock angle, dorsal-sternal diameter, perimeter of anterior cannon bone, angle of shoulder, thoracic perimeter, hip-stifle distance, and angle of croup were the most important traits related with reproductive traits in PRE mares. A multivariate animal model with both morphological and reproductive traits was applied, including age, geographical area, coat color, and average stud size in the decade of the mare’s first foaling. The results indicate that reproductive selection is feasible, and is enhanced by the use of morphological traits, given the moderate to high magnitudes of heritability values in both types of traits, which oscillated between 0.1 (interval between first and second foaling) and 0.95 (height at withers). The resulting genetic parameters were used to develop a series of selection indexes based on morphological or morphological and reproductive combination traits to improve reproductive efficiency traits in PRE mares and thus compute the expected genetic response (**EGR**) for the different strategies. The increase in EGR, when comparing EGR using only reproductive traits as selection criteria vs. using a combined index of both morphological and reproductive traits, oscillated between 4.0% for the age at first foaling to 46.8% for the interval between first and second foaling. In addition, two precocity indexes showed positive EGR when the age at last foaling and the interval between first and second foaling, both with and without morphological traits, were used as selection criteria. Therefore, this analysis reveals that the preselection of reproductive traits based on morphological traits is possible in PRE mares. Ultimately, this knowledge will help breeders achieve genetic progress in reproductive traits, leading to healthier and more successful breeding outcomes in horses.

## Teaser

The incorporation of both morphological and reproductive traits as selection criteria to improve reproductive efficiency in Pura Raza Española mares significantly amplifies genetic responses for reproductive efficiency.

## Introduction

There is a long history of selection by horse breeders, dating back hundreds of years, and it has played a significant role in the evolution and development of the current horse breeds. Through selective breeding, humans have been able to enhance and refine the natural abilities and qualities of horses, creating a diverse range of breeds that are well-suited to different tasks and purposes. The main purpose of horse selection is to improve specific traits like speed, agility, strength, conformation, temperament, and other desirable characteristics that are valued in different equine disciplines, such as racing, dressage, jumping, and showing (Koenen et al., 2004; Thorén-Hellsten et al., 2006). However, reproductive efficiency has received very little attention in horse breeding, including in the Pura Raza Española horse (**PRE**), when compared to other livestock species. Fertility directly impacts the ability of a mare to conceive, carry a foal to term, and give birth to a healthy foal, and it has a crucial impact on population management and determines the economic efficiency of the entire production system (Stott et al., 1999; Langlois and Blouin, 2004; Gómez et al., 2020). Moreover, successful reproduction is essential for maintaining a healthy and profitable equine farm, as the birth of a foal represents not only the continuation of the bloodline, but also a significant investment in time and resources.

Fertility can be affected by several factors, such as genetics, age, nutrition, and management practices (Langlois and Blouin, 2004; Gómez et al., 2020). Therefore, it is essential to manage the reproductive function of mares to maximize their reproductive potential. The use of advanced reproductive technologies, such as artificial insemination and embryo transfer, has allowed horse breeders to increase reproductive efficiency and genetic improvement in their breeding programs (Azcona et al., 2020) and overcome some of the challenges associated with infertility. Nevertheless, many aspects can be improved, and there is no generally established routine methodology (such as using detailed records of reproductive events, including mating/artificial insemination dates and any reproductive health issues), despite the great importance of successful selection in improving the genetic potential of mares (Taveira and Dias, 2007; Gómez et al., 2020; Perdomo-González et al., 2021).

Numerous studies have established associations between morphology and reproductive efficiency in various domestic species, including cattle (Pérez-Cabal et al., 2006; Strapák et al., 2011; Williams et al., 2022), pigs (Bohlouli et al., 2023), sheep (Pourlis, 2011), and goats (Khandaker et al., 2017; Ziadi et al., 2021). Although many studies have explored the relationship between conformation and functionality (Gómez et al., 2021) or functional performance in sports (Sánchez-Guerrero et al., 2016), leading to the development of indirect selection indexes (Sánchez-Guerrero et al., 2017), there is currently a severe lack of research analyzing the association between conformation and reproductive performance in horses.

The PRE horse is a major horse breed that originates from Spain, has become an iconic symbol of Spanish culture and tradition and has played an important role in Spanish economy, history, and folklore (Poyato-Bonilla et al., 2022). Its distinctive appearance and noble temperament have made it a favorite of artists and writers throughout the centuries. This breed is recognized for its remarkable versatility, excelling in various equestrian disciplines, such as Dressage, Spanish High School Riding, Doma Vaquera, and working equitation, thanks to its exceptional riding and working equitation. Moreover, it is often praised for its stunning beauty, grace, athleticism, and striking appearance (Sánchez-Guerrero et al., 2014). Today, there are 268,425 active PRE horses (MAPA, 2023), distributed across 65 countries over five continents (Solé et al., 2019), managed by the Real Asociación Nacional de Criadores de Caballos de Pura Raza Española (ANCCE). Since its inception in 1912, the PRE studbook has maintained a closed breeding program, only permitting the registration of horses with registered parents. In addition, in the early 1980s, affiliation tests were conducted on PRE horses using various molecular techniques, which have allowed for a comprehensive pedigree with over 40 years of established parental information (Perdomo-González et al., 2021). The PRE horse is considered a genetically pure breed, and efforts are being made to preserve and protect its unique characteristics. In many PRE herds, the main objective is reproduction, to obtain foals that are usually sold at an early age. However, many of these breeders usually follow a line breeding system (Gómez et al., 2009), which can cause inbreeding depression problems in the animals, lowering the reproductive efficiency (Perdomo-González et al., 2021).

The PRE Breeding Program is a set of regulations approved in 2003 by the Directorate-General for Agricultural Productions and Markets of the Spanish Government which establish the basis of the basic requirements for regulating selection schemes and performance controls for the genetic evaluation of pure-bred Equidae. Up until now, the PRE Breeding Program has only included dressage and conformation traits as breeding objectives. Therefore, determining the correlation between morphological traits, evaluated using measurement systems and the linear morphological classification system, and reproductive efficiency parameters could prove to be of great interest to improve and optimize the early selection of reproductive efficiency.

The objective of this study was, therefore, to determine the relationship between morphology and reproductive traits in the PRE horse as a basis for designing an early selection index to improve reproductive performance, using only conformation records or using a mixed strategy including both reproductive efficiency variables and some related conformation variables as preselection criteria.

## Material and Methods

### Data set

The total PRE phenotypic data set, provided by the ANCCE, includes 29 biometric and 9 linear traits from more than 158,000 horses (one record per trait per horse, both males and females) collected from 2008 to 2022. The biometric traits were systematically collected in official breed controls, which all PRE horses are obliged to pass before being included in the breeders register of the official studbook, by trained evaluators using standard measuring sticks and non-elastic measuring tape, as described by Sánchez-Guerrero et al. (2016). All the measurements were taken from the left side of the horse while it was standing on a firm, flat surface, assuming a natural position. The linear traits were collected using the linear scoring system in horses to refine the morphological trait definitions and increase the objectivity of the trait assessments used in the PRE Breeding Program from 2008 (Valera et al., 2010). Reproductive information was obtained from the PRE studbook, which includes a total of 383,328 animals (196,017 females) born from the early 1900s up to 2023. The following reproductive traits were calculated, as described in Perdomo-González et al. (2021): age at first foaling (**AFF**) in months, age at last foaling (**ALF**) in months, average interval between first and second foaling (**I12**) in months, average interval between foaling (**AIF**) in months, total number of foalings (**FN**) and total reproductive efficiency (**RE**). RE is the number of total foalings relative to the optimal number of foalings the mare could have during her entire life. The optimal number of foalings was calculated individually for each mare on the age she first foaled and the age she was culled from the breeding herd or the age at her last foaling in the case she has not finished her productive life, assuming an optimal foaling frequency of one per year from the initial foaling. To increase the accuracy of the data, were selected mares born after 1970 (the year when paternity controls began in the PRE) who had their first foal on a farm specialized in foal production (producing over 12 foals per year). For further refinement of our selection criteria, mares used for leisure or sport were excluded, leaving those who had their first foal between 4 and 7 years old, an interval between first and second foaling equal to or below 5 years, an average interval between foaling greater than 9 months, and an interval between the last and penultimate foaling below 5 years.

### Statistical and genetic analysis

All the morphological and reproductive traits were first analyzed using REML univariate models to obtain preliminary estimates of variance components and breeding values. Univariate animal models equal to those described in Sánchez et al. (2013) were applied for the total morphological traits dataset, with males and females. The model included evaluation age (three classes: less than 4 years; between 4 and 7 years; more than 7 years), sex (two classes: male and female), geographical area of the stud (seven classes: Spain, the rest of Europe, the United States and Canada, South America, Africa and Arabian Peninsula, and the rest of the world) and the coat color (four classes: gray, bay, black, and chestnut) as fixed effects after their significance was determined using a general linear model (GLM) procedure (results in the Supplementary Material). The equation in matrix notation for the morphological model was:

y=Xb+Zu+e , and it contained:


$$\begin{array}{l} \left(\begin{array}{l} u\;e\; \end{array}\right)∼\;N\left(\left[\begin{array}{l} 0\;0\; \end{array}\right],\left[\begin{array}{lll} A\sigma {\;}_{u}^{2} & 0\;0 & I\sigma {\;}_{e}^{2}\; \end{array}\right]\right), \end{array}$$

where **y** is the vector of observations, **X** the incidence matrix of systematic effects, **Z** the incidence matrix of animal genetic effects, **b** the vector of systematic effects, **u** the vector of direct animal genetic effects, **e** the vector of residuals, $\sigma {\;}_{u}^{2}$ the direct genetic variance, $\sigma {\;}_{e}^{2}$ the residual variance, **I** an identity matrix, and **A** the numerator relationship matrix.

For the reproductive traits, the animal model used was the same as those described in Perdomo-González et al. (2021). Models included the mare’s coat color (four classes: gray, bay, black, and chestnut), geographic stud zone (three classes: Spain, the rest of Europe, and the rest of the world), and average stud size in the decade of the mare’s first foaling (15 classes, except for ALF, with 12 classes) as fixed effects after their significance was determined using a general linear model (GLM) procedure (results in the Supplementary material). The average stud size in the decade of the mare’s first foaling is the result of the combination of the stud size and the respective decade of the mare’s first foaling. The stud size is directly related to the management system and the productive orientation. Small studs, with less than three foals born per year, have a traditional management system, medium studs, between three and nine foals born per year, have a mixed production system (still traditional but more specialized) and big studs, with more than nine foals born per year, have an intensive production system with modern reproductive technology as artificial insemination or embryo transfer. In addition, ALF was included in the FN, AIF, and RE models as a covariate (linear and quadratic) due to its direct relationship with the mare’s reproductive period. The moment when mares have their first foaling is a breeder’s decision rather than a biological issue, while the age at last foaling is directly related to the mare’s reproductive efficiency and aptitude. The equation in matrix notation for the reproductive model was: y=Xb+Zu+e, with the vectors and matrices as described above.

The pedigree information for genetic evaluation was collected from the PRE official stud-book. At least four complete generations of all the horses with records were included in the pedigree file, making a total of 170,442 for morphology and 344,707 animals for reproductive traits.

The estimated breeding values (**EBV**) for the morphological and reproductive traits of the coincident animals (animals genetically evaluated for both morphological and reproductive traits) were used to perform a partial least squares (**PLS**) analysis, which is a multivariate statistical analysis technique used to identify the underlying relationships between sets of variables. The estimated breeding values of a total of 169,142 PRE horses were used for the PLS analysis with the PLS package for R. For trait selection, the Wold Criterion (Wold, 1994) was applied, selecting only those variables with a variable importance for projection statistic (**VIP**) greater than one. The VIP metric summarizes a variable’s contribution to the model. If a predictor has a small coefficient (absolute value) and a low VIP value, it may be a suitable candidate for removal. For the index analysis, the morphological traits with the strongest values were selected.

Afterward, to guarantee that the genetic and phenotypic correlations were consistent, that is, to ensure that their covariance matrix was positive and semi-definite, a multivariate analysis was also performed including all the morphological traits selected by the PLS procedure and the six reproductive traits. The combined data set included morphological and reproductive information of a total of 19,758 PRE mares, born from 1970 to 2015. The final multivariate model included age at morphological evaluation (three classes: less than 4 years old; between 4 and 7 years old; over 7 years old), geographical area (three classes: Spain, the rest of Europe, and the rest of the world), coat color (four classes: gray, bay, black, and chestnut) and average stud size in the decade of the mare’s first foaling (12 levels) as fixed effects. The pedigree matrix was formed with the information about 36,798 horses.

All the genetic evaluations were performed using a Bayesian approach via Gibbs sampling using the GIBBSF90 + and POSTGIBSF90 + modules of the BLUPF90 + software (Misztal et al., 2018; Lourenco et al., 2022). The Gibbs sampler was run for 250,000 rounds, with the first 50,000 considered as burn-in, after which every sample was saved for later analysis. Posterior means and standard deviations were calculated to obtain estimates of (co)variance components. Convergence of the posterior parameters was assessed by visual inspection of trace plots of posterior distributions generated by the Coda R package (Plummer et al., 2006).

### Expected genetic response

The classic selection indexes theory (Hazel and Lush, 1943) and its adaptation for the use of estimated breeding values (Gutiérrez et al., 2014) was used to calculate the expected genetic response (**EGR**). The selection objectives for the traits consisted of each reproductive trait independently and all of them together except RE, because it was generated from FN. Three main groups of indexes were developed, using the same selection criteria as the selection objective (type 1), using only the selected morphological traits as selection criteria (type 2), and combining the selected morphological traits and each selection objective as selection criteria (type 3). In addition, two more indexes were developed as precocity indexes, in which the selection objective was RE, first based on the combination of AFF and I12 as selection criteria (type 4) and then combining the selected morphological traits, AFF and I12 as selection criteria (type 5). The genetic responses using different objectives/criteria were computed and compared. In cases where just one reproductive trait was used as a selection objective, the desired economic weight vector (**p´**) was 100% (or −100% in the case of AFF, I12, and AIF, when the final selection objective was to reduce them). When the selection objectives were all the reproductive traits together except RE, the **p´** vector was specially adapted to the nature of each trait, with −10%, 25%, 10%, −25%, and 45% for AFF, ALF, I12, AIF, and FN, respectively.

The weights used in vector **b**ʹ for weighting the EBV on **v** were obtained using the formula **b´** = **p´C´G**^−1^, where **Cʹ** is the covariance matrix between the objectives in vector **u** and the EBV used as criteria in vector **v**, **G** is the (co)variance matrix for the selection objectives **u**. The EBV were used as independent variables to estimate the genetic response. The **Cʹ** and **G** matrices were obtained from the genetic parameters by assuming all the additive genetic variances to be unity and therefore using the same, identical genetic scale ($\sigma {\;}_{u1}^{2}$ = $\sigma {\;}_{u2}^{2}$ = $\sigma {\;}_{u3}^{2}$ …= $\sigma {\;}_{uk}^{2}$ =1), for all of them, where $\sigma {\;}_{uk}^{2}$ is the additive genetic variance of trait *k*. Note that the coefficients in **b** varied when considering different criteria and/or objectives, and matrices **C** and **G** also changed. When objective and criteria are the same traits, the (co)variance matrix between the objectives and the criteria **C** becomes a genetic additive (co)variance matrix, in which the diagonals are equal to one (Gutiérrez et al., 2014). Off-diagonal elements are the genetic correlations between objectives and criteria, given that $r_{u_{k}u_{i}}=\frac{{\sigma \;}_{u_{k}u_{i}}}{\sqrt{\sigma {\;}_{u_{k}}^{2}}\sigma {\;}_{u_{i}}^{2}}$, where $r_{u_{k}u_{i}}$ is the genetic correlation between the traits *k* and *I* and $\sigma {\;}_{u_{k}}^{2}=1$ for any trait, thus becoming ${\sigma \;}_{u_{k}u_{i}}=\;r_{u_{k}u_{i}}$ and **C**´:


$$\begin{array}{l} \begin{array}{lll} & C^{\prime }=Var(u)=\left[\begin{array}{lll} \begin{array}{lll} \sigma {\;}_{u_{1}}^{2} & {\sigma \;}_{u_{1}u_{2}}\;{\sigma \;}_{u_{2}u_{1}} & \sigma {\;}_{u_{2}}^{2}\; \end{array} & \begin{array}{lllll} {\sigma \;}_{u_{1}u_{3}} & \ldots & {\sigma \;}_{u_{1}u_{m}}\;{\sigma \;}_{u_{2}u_{3}} & \ldots & {\sigma \;}_{u_{2}u_{m}}\; \end{array}\;\begin{array}{llll} \ldots & \ldots \;\ldots & \ldots \;{\sigma \;}_{u_{m}u_{1}} & {\sigma \;}_{u_{m}u_{2}}\; \end{array} & \begin{array}{lllllll} \ldots & \ldots & \ldots \;\ldots & \ldots & \ldots \;{\sigma \;}_{u_{m}u_{3}} & \ldots & \sigma {\;}_{u_{m}}^{2}\; \end{array}\; \end{array}\right]=\; & \left[\begin{array}{lll} \begin{array}{lll} 1 & r_{u_{1}u_{2}}\;r_{u_{2}u_{1}} & 1\; \end{array} & \begin{array}{lllll} r_{u_{1}u_{3}} & \ldots & r_{u_{1}u_{m}}\;r_{u_{2}u_{3}} & \ldots & r_{u_{2}u_{m}}\; \end{array}\;\begin{array}{llll} \ldots & \ldots \;\ldots & \ldots \;r_{u_{m}u_{1}} & r_{u_{m}u_{2}}\; \end{array} & \begin{array}{lllllll} \ldots & \ldots & \ldots \;\ldots & \ldots & \ldots \;r_{u_{m}u_{3}} & \ldots & 1\; \end{array}\; \end{array}\right]\; \end{array} \end{array}$$

As **G** and **C**ʹ are directly dependent on the genetic parameters, these matrices can be derived directly from genetic parameters to build the desired index. When the criteria are not the same, then the **C**ʹ matrix is not square, and each element is the genetic correlation between two traits. To compare the genetic indexes, the genetic responses for each one has been obtained by weighting, for each of the traits, all those responses obtained in the correlated selected traits including their own direct genetic self-response. Thus, assuming the EBV are not known for certain, and under the assumption stated above about all the additive genetic variances being one, the direct genetic response would be calculated by the selection intensity (**I)** reduced by the accuracy of the EBV. The correlated response would be the genetic correlation times the selection intensity reduced by the accuracy of the EBV. Assuming that all individuals have the same amount of information, this accuracy is proportional to the square root of heritability of the trait used as a criterion. Gathering this information into a matrix, the cumulated genetic responses will be obtained by:


$$\begin{array}{l} t=b\overset{´}{\;}TI=b\overset{´}{\;}\;=\;\left[\begin{array}{lll} \begin{array}{lll} h_{1} & h_{1}r_{u_{1}u_{2}}\;h_{2}r_{u_{2}u_{1}} & h_{2}\; \end{array} & \begin{array}{lllll} h_{1}r_{u_{1}u_{3}} & \ldots & h_{1}r_{u_{1}u_{m}}\;h_{2}r_{u_{2}u_{3}} & \ldots & h_{2}r_{u_{2}u_{m}}\; \end{array}\;\begin{array}{llll} \ldots & \ldots \;\ldots & \ldots \;h_{k}r_{u_{m}u_{1}} & h_{k}r_{u_{m}u_{2}}\; \end{array} & \begin{array}{lllllll} \ldots & \ldots & \ldots \;\ldots & \ldots & \ldots \;h_{k}r_{u_{m}u_{3}} & \ldots & h_{k}\; \end{array}\; \end{array}\right]\; \end{array}$$

where each Ʃ $t_{jk}$ is the cumulated genetic response in the trait *k*. Therefore, genetic responses *t* were obtained for each trait when selection was based on indexes that weighted the breeding values for the traits of interest. When the criteria are not the same, then objectives **T** matrix is not square, but each element $t_{jk}$ is the genetic correlation between traits *j* and *k*. For the comparison of computed responses, a selection intensity of one was assumed, because this will serve as a constant, leading to comparable relative results.

## Results

Table 1 shows the descriptive statistic for the 29 biometric, 9 linear, and 6 reproductive traits analyzed in the PRE mares. The number of animals with phenotype records ranges between 3,600 (*height of substernal hollow*) and 19,758 (AFF, FN, RE), but for most traits there are close to 5,000 records. The biometric traits mean values oscillate between 8.1 cm for the *height of withers* and 192.4 cm for *thoracic perimeter*, while the linear traits mean values oscillate between 3.9 and 5.3, for *direction of hind hoof*, and *angle of knee front view*, respectively. The CV of most biometric traits is low, except for *angle of croup* (25.8%) and *height of withers* (23.4%), whereas most linear traits show a higher CV, oscillating between 12.6% for *angle of knee side view* and 24.7% for *dorsal-lumbar line*. In addition, the average mean values for reproductive traits were 57.6 months, 141.8 months, 17.5 months, 18.7 months, 5.1 foals, and 45.0% for AFF, ALF; I12, AIF, FN, and RE, respectively, and their CV oscillated between 17.4% (AFF) and 69.9% (FN).

**Table 1.** Descriptive statistics of 38 morphological and 6 reproductive traits in the analyzed Pura Raza Española mares with reproductive information

			*N*	Mean	SD	Min	Max	CV (%)
Morphological traits	Biometric traits, cm	Height at withers	4,186	160.87	4.72	150	178	2.93
		Height of withers	4,790	8.13	1.90	1	19	23.40
		Height at lowest point of withers	4,125	152.79	4.74	140	174	3.10
		Height at point of croup	4,128	160.00	4.64	140	178	2.90
		Height of substernal hollow	3,600	78.35	4.70	60	111	6.00
		Scapular‑ischial length	18,608	159.89	5.34	131	190	3.34
		Proportionality index	4,177	99.63	2.24	90	108	2.24
		Length of head	4,782	61.91	3.14	35	80	5.07
		Width of head	4,780	23.19	1.64	15	35	7.09
		Length of neck	4,782	74.51	4.58	50	90	6.15
		Width of chest	18,671	42.20	3.57	22	63	8.45
		Length of shoulder	8,749	66.34	3.94	41	86	5.94
		Length of forearm	4,822	46.66	3.65	31	77	7.81
		Dorsal-sternal diameter	18,770	73.99	3.90	42	100	5.27
		Bi-costal diameter	4,077	42.54	7.06	25	77	16.60
		Length of loin	4,824	31.24	4.22	14	50	13.51
		Length of mouth	4,782	9.14	1.11	6	14	12.20
		Length of back	4,830	30.56	4.53	18	50	14.82
		Width of croup	4,291	54.22	3.40	23	70	6.27
		Length of croup	4,828	53.29	2.99	38	66	5.61
		Hip-stifle distance	4,824	50.01	4.43	30	64	8.85
		Buttock-stifle distance	4,821	51.23	2.74	30	65	5.36
		Length of leg	4,814	52.49	3.90	31	69	7.43
		Length of buttock	4,820	45.81	4.52	31	65	9.86
		Thoracic perimeter	18,143	192.41	9.13	155	225	4.74
		Perimeter of knee	18,197	31.72	1.51	18	45	4.76
		Perimeter of anterior cannon bone	16,586	20.08	1.24	10	34	6.16
		Angle of shoulder	4,604	55.68	6.52	14	80	11.70
		Angle of croup	3,799	21.07	5.42	2	53	25.75
	Linear traits, classes	Angle of knee front view	4,853	5.27	0.67	1	8	12.72
		Direction of fore hoof	4,385	5.09	0.90	1	9	17.59
		Angle of knee side view	4,854	5.22	0.66	1	9	12.63
		Dorsal-lumbar line	4,852	4.54	1.12	1	9	24.66
		Lateral hock angle	4,852	5.10	0.92	2	9	18.01
		Angle of hock rear view	4,854	4.04	0.85	1	7	21.08
		Direction of hind hoof	4,384	3.86	0.76	1	9	19.77
		Posterior tendon development	4,853	4.81	1.00	1	8	20.78
		Muscular development	4,847	4.71	1.13	1	9	23.91
Reproductive traits		Age at first foaling, months	19,758	57.63	10.04	45	84	17.43
		Age at last foaling, months	16,620	141.76	53.95	55	420	38.06
		Interval between first and second foaling, months	16,620	17.54	8.91	10	60	50.82
		Average interval between foaling, months	16,620	18.74	6.97	9	110	37.16
		Foaling number	19,758	5.08	3.55	1	21	69.90
		Reproductive efficiency, %	19,758	44.99	17.49	5	106	38.87

After the PLS analysis, eight morphological traits have shown a VIP value higher than one (Table 2). The traits with higher VIP values were *height at withers* (**HatW**, VIP = 2.5) and *lateral hock angle* (**LHA**, VIP = 2.1) while for other traits, *dorsal-sternal diameter* (**DSD**), *hip-stifle distance* (**HSD**), *thoracic perimeter* (**TP**), *perimeter of anterior cannon bone* (**PCB**), *angle of shoulder* (**AS**), and *angle of croup* (**AC**), the VIP values ranged from 1.0 to 1.9.

**Table 2.** Partial least squares analysis

Morphological traits	Importance	VIP
Height at withers	1	2.514664
Lateral hock angle	2	2.118650
Dorsal-sternal diameter	3	1.858410
Perimeter of anterior cannon bone	4	1.597397
Angle of shoulder	5	1.359581
Thoracic perimeter	6	1.262050
Hip-stifle distance	7	1.163304
Angle of croup	8	1.027496

Summary of the most relevant morphological traits for reproductive traits in Pura Raza Española mares (VIP > 1).

Variable importance for projection statistic (VIP).

Table 3 shows the heritability and genetic correlation values between and within morphological and reproductive traits from the multivariate model. Heritability values ranged between 0.27 (LHA) and 0.95 (HatW) for morphological traits, and between 0.10 (I12) and 0.27 (FN) for reproductive traits. Genetic correlations between morphological traits were mostly positive, with positive values oscillating from <0.01 (AC-LHA) to 0.71 (HatW-DSD), while negative values oscillated between −0.03 (HatW-AS) and −0.18 (HSD-AC). The highest negative genetic correlation values between reproductive traits were found between I12 and AIF with FN and RE, −0.53 (I12-FN), −0.46 (I12-RE), −0.40 (AIF-FN), and −0.59 (AIF-RE), with the highest positive values being for ALF-FN (0.95), I12-AIF (0.87), and FN-RE (0.40). Over half the genetic correlation values between morphological and reproductive traits were higher than 0.10 in absolute value. The highest positive value was 0.11 (AC-AIF and DSD-RE) and the highest negative values were −0.47 (HSD-ALF) and −0.46 (HSD-FN).

**Table 3.** Heritabilities (diagonal) and genetic correlations (above diagonal) within and between most relevant morphological traits and the reproductive traits in Pura Raza Española mares

	Morphological traits								Reproductive traits					
	HatW (PSD)	DSD (PSD)	HSD (PSD)	TP (PSD)	PCB (PSD)	AS (PSD)	AC (PSD	LHA (PSD)	AFF (PSD)	ALF (PSD)	I12 (PSD)	AIF (PSD)	FN (PSD)	RE (PSD)
HatW	0.95 (0.006)	0.71 (0.016)	0.39 (0.027)	0.60 (0.015)	0.57 (0.019)	−0.03 (0.028)	−0.11 (0.031)	−0.15 (0.035)	0.01 (0.026)	−0.25 (0.024)	0.07 (0.035)	0.02 (0.032)	−0.23 (0.024)	−0.04 (0.032)
DSD		0.38 (0.012)	0.17 (0.038)	0.59 (0.021)	0.46 (0.028)	0.01 (0.048)	−0.11 (0.041)	0.19 (0.046)	<0.01 (0.036)	−0.21 (0.033)	−0.06 (0.053)	0.01 (0.049)	−0.19 (0.032)	0.11 (0.044)
HSD			0.61 (0.033)	0.17 (0.039)	0.31 (0.043)	−0.12 (0.060)	−0.18 (0.050)	−0.14 (0.066)	0.15 (0.053)	−0.47 (0.046)	0.16 (0.078)	0.01 (0.079)	−0.46 (0.045)	−0.16 (0.067)
TP				0.50 (0.036)	0.49 (0.025)	0.07 (0.044)	−0.07 (0.037)	−0.16 (0.049)	−0.01 (0.036)	−0.05 (0.030)	−0.10 (0.048)	−0.14 (0.047)	−0.01 (0.030)	0.10 (0.043)
PCB					0.41 (0.014)	0.15 (0.050)	−0.04 (0.043)	0.03 (0.059)	<0.01 (0.038)	−0.20 (0.034)	0.03 (0.054)	0.01 (0.053)	−0.18 (0.034)	0.10 (0.048)
AS						0.53 (0.046)	0.20 (0.056)	−0.04 (0.083)	−0.04 (0.067)	−0.12 (0.060)	0.16 (0.090)	0.09 (0.091)	−0.10 (0.061)	0.03 (0.080)
AC							0.65 (0.039)	<0.01 (0.070)	<0.01 (0.056)	−0.26 (0.050)	0.12 (0.075)	0.11 (0.070)	−0.24 (0.051)	−0.05 (0.066)
LHA								0.27 (0.033)	−0.16 (0.069)	−0.13 (0.064)	0.04 (0.093)	−0.10 (0.093)	−0.05 (0.065)	0.02 (0.085)
AFF									0.21 (0.015)	−0.12 (0.043)	0.34 (0.066)	0.36 (0.064)	−0.35 (0.040)	−0.15 (0.060)
ALF										0.26 (0.012)	−0.33 (0.059)	−0.13 (0.057)	0.95 (0.006)	0.22 (0.046)
I12											0.10 (0.011)	0.87 (0.023)	−0.53 (0.049)	−0.46 (0.064)
AIF												0.13 (0.016)	−0.40 (0.048)	−0.59 (0.053)
FN													0.27 (0.012)	0.40 (0.039)
RE														0.12 (0.011)

Indexes developed: using the same selection criteria as the selection objective (type 1), using only the selected morphological traits as selection criteria (type 2) and combining the selected morphological traits and each selection objective as selection criteria (type 3). Posterior standard deviation (PSD), height at withers (HatW), dorsal-sternal diameter (DSD), hip-stifle distance (HSD), thoracic perimeter (TP), perimeter of anterior cannon bone (PCB), angle of shoulder (AS), angle of croup (AC), lateral hock angle (LHA), age at first foaling (AFF), age at last foaling (ALF); average interval between first and second foaling (I12), average interval between foaling (AIF), total number of foalings (FN), and reproductive efficiency (RE).

Table 4 shows the EGR for each reproductive trait for each designed index and their respective increase in response with respect to the index type 1 (using the same selection criteria as the selection objective, fixed as a relative response of all 100%). When each reproductive trait is analyzed as a selection objective independently, the highest selection response in type 1 indexes can be seen for FN, ALF, and AFF (0.291, 0.263, and −0.241, respectively), but RE and AIF and I12 also exhibit favorable responses (0.146, −0.160, and −0.104, respectively). Type 2 selection indexes also obtained favorable results for each reproductive trait; nevertheless, in all cases, the selection response was lower than the EGR obtained in type 1 indexes. It is noteworthy that the EGR for ALF with only the morphological traits as selection criteria (type 2) was only 21.6% lower than the response obtained using only the ALF as selection criteria (type 1). For FN and I12, type 2 indexes EGR are 35.6% and 42.2% lower than the results for type 1 indexes, respectively, while those for RE, AIF, and AFF type 2 indexes EGR were 70.7%, 77.7%, and 90.6% lower than the results for type 1 indexes, respectively. On the other hand, when each reproductive trait is analyzed with type 3 indexes (combining morphological traits and the respective selection objective), all the EGR values show the highest values when compared with the type 1 response values. In this context, the reproductive traits with the highest increase in response were I12, ALF, and FN, with response values of 46.8%, 28.3%, and 16.4% higher than in the type 1 index. Also, increases in response of 10.14%, 11.59%, and 4.49% higher when compared to type 1 indexes were obtained for RE, AIF, and AFF, respectively. Identical increases in response can be observed with the type 3 selection index when comparing all the reproductive traits except RE together as selection objectives with those obtained for each reproductive trait independently. It is worth mentioning that when type 2 indexes are compared, AFF, I12, and AIF obtained a better increase in response when all the reproductive traits except RE selection objective are used.

**Table 4.** Expected genetic responses and the increase in response respect to index 1 (in parentheses) for the selection indexes associated with reproductive traits in Pura Raza Española mares

Objective	Index type	AFF	ALF	I12	AIF	FN	RE
RE	1						0.146 (100)
	2						0.043 (29.44)
	3						0.160 (110.14)
	4						0.031 (21.27)
	5						0.050 (34.35)
FN	1					0.291 (100)	
	2					0.184 (63.43)	
	3					0.338 (116.36)	
AIF	1				−0.160 (100)		
	2				−0.035 (22.32)		
	3				−0.178 (111.59)		
I12	1			−0.104 (100)			
	2			−0.060 (57.83)			
	3			−0.152 (146.77)			
ALF	1		0.263 (100)				
	2		0.206 (78.39)				
	3		0.337 (128.27)				
AFF	1	−0.241 (100)					
	2	−0.022 (9.41)					
	3	−0.251 (104.49)					
All reproductive traits except RE	1	−0.086 (100)	0.183 (100)	−0.077 (100)	−0.083 (100)	0.218 (100)	
	2	−0.026 (31.06)	0.135 (74.17)	−0.052 (67.66)	−0.030 (36.59)	0.133 (61.17)	
	3	−0.090 (104.49)	0.235 (128.27)	−0.113 (146.77)	−0.092 (111.59)	0.254 (116.36)	

Increase in response is calculated respect to index 1 fixed as a relative response of the 100%. Age at first foaling (AFF), age at last foaling (ALF); average interval between first and second foaling (I12), average interval between foalings (AIF), total number of foalings (FN), and reproductive efficiency (RE). Index type 1: each reproductive trait is analyzed as a selection objective independently; index type 2: only morphological traits were used as selection criteria; index type 3: combining morphological traits and the respective selection objective as selection criteria; index type 4: combining AFF and I12 as selection criteria; index type 5: combining the selected morphological traits, AFF and I12 as selection criteria.

Finally, type 4 and 5 indexes were designed to evaluate the increase in response for the RE precocity selection (combining AFF and I12 as selection criteria in type 4 and adding the selected morphological traits to the two previous traits in type 5). Both models showed considerable response values, indicating 21.27% (type 4) and 34.35% (type 5) in response, when compared with the type 1 index. The difference in increase between type 5 and type 2 indexes for RE objective is particularly noteworthy, with the inclusion of AFF and I12 causing an increase of almost 5% in the selection response. Nevertheless, the use of AFF and I12 alone as selection criteria (type 4) does not improve the selection response for RE when compared to the use of morphological traits exclusively (type 2 index).

## Discussion

The genetic evaluation of reproductive efficiency in mares is a complex process, mainly because evaluating their fertility can be, for several reasons, challenging. Firstly, fertility is a multifaceted trait that is influenced by genetic and environmental factors, and it can be tricky to separate the effects of each factor. Moreover, influential factors such as age, nutrition, and management practices vary between individuals and breeders (Langlois and Blouin, 2004; Gómez et al., 2020). Secondly, it can prove hard to obtain accurate and reliable measurements of fertility, particularly in extensive breeding systems, in addition to the fact that reproductive examinations and evaluations can be costly and time-consuming, particularly in large populations of mares.

On the other hand, reproductive traits are also influenced by interactions between multiple genes and genetic pathways (Li et al., 2020; Laseca et al., 2022), which can make it difficult to identify specific genes or genetic markers associated with globally assessed traits. As a result, the heritability values of reproductive traits are typically lower than those for morphology, conformation, or sport traits. Despite these challenges, there are ongoing efforts to improve the response of reproductive traits to selection through better recording of reproductive information, selective breeding, and the use of advanced genetic technologies. In addition, the establishment of indirect selection actions for reproductive efficiency from morphological records, similar to those already implemented in the PRE horses for dressage (Sánchez-Guerrero et al., 2014) could be highly effective.

Direct measurement and the linear scoring system have given us a better understanding of the biological relationship between conformation traits. Many studies have analyzed the genetic parameters and correlations between a large number of morphological and conformational traits in different horse breeds. One study analyzing PRE horse morphology differentiating between genders (Sánchez-Guerrero et al., 2016) showed similar or slightly higher mean values than those reported here for *height at withers* (154.7, 156.1, and 157.9), *width of chest* (42.4, 42.8, and 41.9), *dorsal-sternal diameter* (72.2, 73.6, and 73.9), *thoracic perimeter* (190.0, 192.1, and 190.9), *perimeter of knee* (31.0, 31.1, and 31.2), *perimeter of anterior cannon bone* (19.6, 19.7, and 20.2), for mares in three different time periods: mares born before 1990, mares born from 1990 to 2001 and mares born from 2002 to 2013, respectively, to illustrate the morphological evolution over time. Similarly, slightly lower mean values were reported by Gomez et al. (2021) for adult female PRE horse for *scapular-ischial length* (159.60), *length of head* (61.55), *width of head* (23.26), *length of neck* (74.23), *length of shoulder* (65.87), *length of croup* (52.87), *hip-stifle distance* (49.6), *thoracic perimeter* (191.14), *perimeter of knee* (31.27), *perimeter of anterior cannon bone* (19.92), and *width of chest* (40.78).

The reproductive efficiency traits examined in this study were chosen for their recognized importance in assessing the reproductive performance of mares (Gómez et al., 2020). AFF and ALF mark the beginning and end, respectively, of an individual’s reproductive lifespan. AIF and I12 measure the time span between foaling events, providing indirect indicators of fertility. FN directly corresponds to the duration of an animal’s reproductive lifespan. Moreover, RE is utilized to assess fertility efficiency at any specific moment. The mean values for reproductive efficiency traits were similar to those obtained by Perdomo-González et al. (2021) for the same breed, although AFF, I12, and FN showed slightly lower values (57.6, 17.5, and 5.0, respectively), and AIF, ALF, and RE mean values were slightly higher (18.8, 169.7, and 45.5, respectively). Concurrently, our findings concerning the average values of reproductive traits align closely with those of Gómez et al. (2020) in the same population. Their study encompassed an analysis of reproductive parameters in PRE mares and seven additional Spanish horse breeds, namely Arab Horse, Purebred Menorca Horse, Spanish Sport Horse, Anglo-Arab Horse, Spanish Trotter Horse, Jaca Navarra, and Burguete. Those authors reported slightly higher values for AFF (64.6), I12 (20.0), and AIF (20.9) in PRE mares, while observing lower values for ALF (125.7). This reflected the PRE mares’ exceptional early development compared to other Spanish breeds, as they displayed the lowest values for all the assessed traits within the analyzed breeds. Conversely, Sabeva and Apostolov (2011) documented a higher average number of foals in Arabian broodmares (6.4) than in our study.

After the PLS analysis, those traits with a VIP value higher than one were selected. Specifically, seven biometric traits and one linear trait were selected: *height at withers* (HatW), *lateral hock angle* (LHA), *dorsal-sternal diameter* (DSD), *perimeter of the cannon bone* (PCB), *angle of shoulder* (AS), *thoracic perimeter* (TP), *hip-stifle distance* (HSD), and *angle of croup* (AC). These measurements play a crucial role in horse breeding by providing objective criteria for evaluating conformation, athleticism, soundness, and performance potential (Peña et al., 2009; Valera et al., 2009). Breeders use these measurements to make informed decisions regarding breeding stock selection, improving breed standards, and optimizing the breeding outcomes to produce horses with desirable traits for specific purposes.

The heritability values for the morphological traits were, in general, higher than those previously reported for the PRE horse breed. Sánchez et al. (2013) reported lower heritability values of 0.06, 0.30, 0.15, 0.08, and 0.09 for AS, PCB, AC, HSD, and LHA, respectively, in a population of 12,381 PRE horses, recorded as linear traits in both males and females. Nevertheless, very high results were also found for HatW by Poyato-Bonilla et al. (2020) (0.80), who evaluated inbreeding depression load in a PRE population with both males and females. Lower heritability values have also been reported by Perdomo-González et al. (2022) for HatW, LHA, DSD, AS, TP, HSD, and AC (0.19, 0.10, 0.21, 0.12, 0.41, and 0.21, respectively) in the current population of Pura Raza Menorquina horse, another autochthonous Spanish horse breed, and a breed which is genetically very close to the PRE horse (Negro et al., 2016).

The heritability values for the reproductive traits were, in general, slightly higher than those reported by Perdomo-González et al. (2021), who evaluated inbreeding depression load for AIF (0.10) and RE (0.10), but appreciably higher for AFF (0.16), ALF (0.10), I12 (0.05), and FN (0.08), in a selected population of PRE mares. Gomez et al. (2020) also reported lower values for PRE mares for AFF (0.15), ALF (0.08), and I12 (0.04), but a similar value for AIF (0.14). Relevant differences were also found comparing values to those reported by Karlau et al. (2023) in Criollo Argentino mares for AFF (0.16), ALF (0.19), and RE (0.11). Age at first foaling heritability values have been also analyzed in Arab (0.20), Anglo-Arab (0.16), and Spanish Sport Horses mares (0.32) by Gomez et al. (2020), and in Thoroughbred mares (0.38) by Taveira and Dias (2007), while lower heritability values were described for FN by Wolc et al. (2009) in Warmblood mares (0.12) and Sairanen et al. (2009) in Standardbred (0.01) and Finn horses (0.03). These diverse results are likely due to the need to model fertility using large datasets and quantitative approaches, which are scarce in mares, especially those that directly estimate fertility based on foaling percentage.

In general, the genetic correlations between morphological traits in our study were slightly higher than those obtained by Sanchez-Guerrero et al. (2017) between AS, PCB, AC, HSD, and LHA. The most commonly studied traits seem to be HatW, TP, and PCB. In the Sardinian Anglo-Arab horse, higher genetic correlation values were estimated between HatW-TP (0.71) and HatW-PCB (0.73), while a lower value was estimated between TP-PCB (0.33) by Giontella et al. (2020). Similar results were detected when the same traits were analyzed in the Murgese horse breed by Bramante et al. (2016), who reported higher genetic correlation values between HatW-PT (0.70), TP-PCB (0.70), and HatW-PCB (0.64). The desirable values for the morphological traits are related to the breed prototype established in the breeding program. In this way, traits such as LHA, DSD, PCB, and AS are selected close to the middle range of the biological scale, while higher values are desirable in traits such as HatW, HSD, and AC (Perdomo-González et al., 2023). Genetic correlations between reproductive traits are scarce because they used to be analyzed independently, even when most of them seem to have moderate to high magnitude values, both positive and negative, as shown in this study. Karlau et al. (2023) reported very different genetic correlation values in Criollo Argentino mares of 0.83, −0.55, and −0.86 for AFF-ALF, AFF-RE, and ALF-RE, respectively, which probably implies that if this work can serve as a model for other breeds, its results cannot be directly applied, and studies should be conducted using their own parameters.

There is little information on the genetic relationship between morphological and reproductive traits in mares, considering that the response to selection for morphological traits, which is more common in the equine world, affects reproductive traits, even if they are not being selected. Genetic correlations guide selection decisions, aid in designing effective breeding strategies, predict genetic potential, contribute to our understanding of genetic mechanisms, and facilitate breed-specific goals. Nonetheless, to the best of our knowledge, no data have been reported for genetic correlations between morphological and reproductive traits in other equine breeds. In this study, the highest correlation values were found between ALF and FN with HatW, DSD, HSD, PCB, and AC, all of which were negative and with moderate to high values. Strong, negative correlation values between those traits imply that shorter distances for HatW, DSD, HSD, PCB, and AC will promote a higher age at last foaling and an increase in the total number of foalings the mare can have. From the eight morphological traits selected after the PLS, four are currently included in the selection morphological index for dressage in the Pura Raza Española horse, with higher values for HatW and HSD and lower values for AS and AC most highly sought after. As a result, for example, from the positive correlation values described between I12-HSD (0.16) and I12-AS (0.16), it can be deduced that the shorter distances of HSD and AS will reduce the I12, in the interest of reproductive efficiency; however, PRE breeders are currently selecting for higher HSD values. Moderate genetic correlation was reported in Spanish dairy Holstein cows from the Basque and Navarra Autonomous Regions in Spain by Perez-Cabal et al. (2006), who found a correlation of 0.1 between feet and legs conformation and profit per cow per year (the disparity between the income and costs over the entire productive lifespan of each cow), while the foot angle and rear legs set had a very low correlation. Accordingly, the rear legs set was the locomotion trait that correlated most closely to milk production, while calving interval had higher correlations with foot angle and feet and legs conformation (0.17 and 0.12, respectively). Le et al.(2016) identified a relationship between the effect of leg weakness and poor conformation on fertility loss, analyzing leg conformation in young pigs and sow longevity. In our study, this effect has been evidenced in such important parameters as PCB and LHA, probably because improved leg quality generally results in positive effects on the longevity of the horse and impacts indirectly on the number of offspring. The importance of limb quality in horse locomotion is such that it likely impacts the breeder’s selection of breeding females, aiming to produce more foals from those with superior limb quality. This was also observed in Spanish dairy Holstein cows from the Basque and Navarra Autonomous Regions in Spain by Perez-Cabal et al. (2006), who found that good scores for feet and legs had significative effects on the profit per cow per year. In addition, a genetic parameter estimation study between conformation and reproduction traits analyzed in German local Swabian-Hall landrace sows (Bohlouli et al., 2023) determined that an increase in body height and especially in body length of the sows was associated genetically and phenotypically with an increase in litter size. In our case, the increase in body size associated mainly with the variables HatW, DSD, and TP may be linked to the fact that larger mares have larger foals, which are more viable and more desirable for breeders looking for more precocious animals.

Selecting for productive longevity poses a further challenge, as the true lifespan of an animal remains uncertain until it either dies or becomes unfit for breeding. Consequently, it is of great use to have early indicators that can predict its phenotypic and genetic quality for survival. Linear-type traits, often assessed in young animals, offer a visual evaluation of a wide range of physical characteristics that encompass the expected biological extremes within a particular breed (Berry et al., 2004; Manafiazar et al., 2015). These traits exhibit moderate to high heritability values and have demonstrated significant phenotypic and genetic correlations with longevity measures in Holstein (Kern et al., 2015), Holstein-Friesian (Brotherstone et al., 1998), and Guernsey dairy cows (Cruickshank et al., 2002).

Disregarding genetic and phenotypic correlations between traits may result in unanticipated consequences regarding the correlated response. To address this issue, selection indexes have been devised to integrate multiple diverse traits associated with the ultimate profitability goal. These indexes serve as dynamic tools that can be adjusted to align with the various economic objectives. While selection indexes have long been utilized in dairy cow breeding (Miglior et al., 2005), only a few have been examined and implemented in other species, such as beef cattle (Amer et al., 2001), sheep (Byrne et al., 2010), alpaca (Gutiérrez et al., 2014), and goat populations (Ziadi et al., 2021). In the case of horses, only one study (Sánchez-Guerrero et al., 2017) suggests the possibility of preselecting animals for dressage training based on morphological and linear traits. This study was the first to explore the genetic correlation between morphological and reproductive traits in equine populations, as well as develop selection indexes incorporating these variables in mares.

Given the recognized difficulties in computing realistic economic weights in reproductive traits, indexes such as these are not usually computed. In this study, a method based on the selection index theory by Hazel and Lush (1943), reformulated to use EBV by Gutierrez et al. (2014), was implemented. The moderate to high heritability values observed in the morphological and reproductive traits, coupled with a noticeable enhancement in genetic response when comparing selection indexes based on various criteria (solely reproductive traits, solely morphological traits, or a combination of both), along with the efficacy of the two precocity indexes, present an opportunity to enhance reproductive efficiency in the coming decades. This can be achieved by preselecting PRE horses using early reproductive information in conjunction with their morphological data. As expected, according to our results, the response of a selection index using only morphology would not be as good as direct selection for reproductive traits, although in some cases the correlated response can be very high, as in the case of ALF, with a selection response close to 80% of the genetic response when using only ALF as selection criteria. Our results also reveal a considerable increase in response to the selection when morphological and reproductive traits are included jointly as selection criteria with the objective of increasing the reproductive efficiency, as can be seen for I12, with a selection response close to 50% higher than using only I12 data as selection criteria. Four of the eight selected variables are included in the morphology selection index for dressage. The HatW and the HSD are positively weighted in the index, while the AS and the AC are weighted negatively: in other words, the smaller the angles, the better. Selection for both AS and AC is favorable for reproductive efficiency, as they have negative correlations with ALF and FN (which increases these variables) and positive correlations with I12 and AIF (the lower, the better). Selection for both HatW and HSD favors ALF and FN, and although they have correlations that increase I12 and the overall RE, these correlations are low to moderate magnitude. This means that even when some traits directly selected for performance are indirectly damaging reproductive efficiency, this flaw will only have a minimum impact on it.

Finally, even though the use of a morphological traits index had a relatively lower response, its use could be essential in young animals without any reproductive records with which to carry out an early pre-selection, as precocity indexes have revealed that an early selection of individuals for their reproductive characteristics, even before reproductive information on the animal itself is available, is possible. This holds particular significance due to our knowledge of the high correlation in morphological measurements between young and adult PRE horses (Gómez et al., 2021). Furthermore, breeders of sport horses often allow mares to compete for a few years before transitioning them to breeding, without having any prior knowledge of their reproductive function. The availability of existing morphological information thus presents an early, expeditious opportunity to make informed decisions in this regard.

In summary, ensuring optimal reproductive health and fertility in horses is critical for the success and sustainability of the equine industry. By implementing appropriate breeding management practices and utilizing advanced reproductive technologies, horse breeders can improve their chances of producing healthy, genetically superior foals. Until now, the standard procedures in PRE breeding programs have used morphological and dressage estimated breeding values to select the optimal PRE horses for reproduction. Nevertheless, the results obtained in this study indicate that including morphology improves the selection response for reproductive traits, and although discrete, the use of early selection criteria such as AFF and I12 (especially with the inclusion of morphology) can allow the pre-selection of the best females to use for breeding.

## Supplementary Material

skad409_suppl_Supplementary_Material

## Abbreviations

AC: angle of croup
AFF: age at first foaling
AIF: average interval between foaling
ALF: age at last foaling in months
AS: angle of shoulder
DSD: dorsal-sternal diameter
EBV: estimated breeding values
EGR: expected genetic response
FN: total number of foalings
HatW: height at withers
HSD: hip-stifle distance; I12, average interval between first and second foaling
LHA: lateral hock angle
PCB: perimeter of anterior cannon bone
PLS: Partial Least Squares
PRE: Pura Raza Española
RE: total reproductive efficiency
TP: thoracic perimeter
