# Supplementary material for: Designing an early selection morphological traits index for reproductive efficiency in Pura Raza Española mares
Source: J Anim Sci. 2023 Dec 20;102:skad409. doi: 10.1093/jas/skad409 (PMC10762892; doi:10.1093/jas/skad409)
Supplement: skad409_suppl_Supplementary_Material [file skad409_suppl_supplementary_material.docx]

**Supplementary material.** *P*-values from the General Linear Model for the morphological and reproductive data.

| **Morphological data** | **Coat color** | **Geographic zone** | **Age** | **Sex** |
| --- | --- | --- | --- | --- |
| Height at withers | < 0.001 | < 0.001 | < 0.001 | < 0.001 |
| Height of withers | < 0.001 | < 0.001 | < 0.001 | < 0.001 |
| Height at lowest point of withers | < 0.001 | < 0.001 | < 0.001 | < 0.001 |
| Height at point of croup | < 0.001 | < 0.001 | < 0.001 | < 0.001 |
| Height of substernal hollow | < 0.001 | < 0.001 | < 0.001 | < 0.001 |
| Scapular‑ischial length | < 0.001 | < 0.001 | < 0.001 | < 0.001 |
| Proportionality index | < 0.001 | < 0.001 | < 0.001 | < 0.001 |
| Length of head | 0.608 | < 0.001 | < 0.001 | < 0.001 |
| Width of head | < 0.001 | < 0.001 | < 0.001 | < 0.001 |
| Length of neck | < 0.001 | 0.803 | 0.011 | < 0.001 |
| Width of chest | < 0.001 | < 0.001 | < 0.001 | < 0.001 |
| Length of shoulder | 0.723 | < 0.001 | < 0.001 | < 0.001 |
| Length of forearm | < 0.001 | < 0.001 | < 0.001 | < 0.001 |
| Dorsal-sternal diameter | < 0.001 | < 0.001 | < 0.001 | < 0.001 |
| Bi-costal diameter | < 0.001 | < 0.001 | < 0.001 | < 0.001 |
| Length of loin | < 0.001 | < 0.001 | < 0.001 | < 0.001 |
| Length of mouth | 0.101 | 0.0303 | 0.460 | < 0.001 |
| Length of back | 0.128 | < 0.001 | < 0.001 | < 0.001 |
| Width of croup | < 0.001 | < 0.001 | < 0.001 | < 0.001 |
| Length of croup | < 0.001 | < 0.001 | < 0.001 | 0.457 |
| Hip-stifle distance | 0.0795 | < 0.001 | < 0.001 | < 0.001 |
| Buttock-stifle distance | < 0.001 | < 0.001 | < 0.001 | < 0.001 |
| Length of leg | < 0.001 | 0.005 | < 0.001 | < 0.001 |
| Length of buttock | < 0.001 | < 0.001 | 0.002 | < 0.001 |
| Thoracic perimeter | < 0.001 | < 0.001 | 0.011 | < 0.001 |
| Perimeter of knee | < 0.001 | 0.082 | < 0.001 | < 0.001 |
| Perimeter of anterior cannon bone | < 0.001 | < 0.001 | < 0.001 | < 0.001 |
| Angle of shoulder | 0.230 | < 0.001 | 0.002 | < 0.001 |
| Angle of croup | 0.0387 | < 0.001 | < 0.001 | < 0.001 |
| Angle of knee front view | < 0.001 | < 0.001 | < 0.001 | < 0.001 |
| Direction of fore hoof | 0.030 | 0.001 | < 0.001 | < 0.001 |
| Angle of knee side view | < 0.001 | 0.295 | < 0.001 | < 0.001 |
| Dorsal-lumbar line | < 0.001 | < 0.001 | < 0.001 | < 0.001 |
| Lateral hock angle | < 0.001 | < 0.001 | < 0.001 | < 0.001 |
| Angle of hock rear view | 0.664 | < 0.001 | < 0.001 | < 0.001 |
| Direction of hind hoof | < 0.001 | 0.283 | < 0.001 | < 0.001 |
| Posterior tendon development | < 0.001 | 0.002 | < 0.001 | < 0.001 |
| Muscular development | 0.359 | < 0.001 | < 0.001 | < 0.001 |
| **Reproductive data** | **Coat color** | **Geographic zone** | **Stud size** | **ALF as linear-quadratic** |
| Age at first foaling | < 0.001 | 0.001 | < 0.001 | - |
| Age at last foaling | 0.814 | < 0.001 | < 0.001 | - |
| Interval between first and second foaling | < 0.001 | 0.048 | < 0.001 | - |
| Average interval between foaling | < 0.001 | 0.019 | < 0.001 | < 0.001 |
| Foaling number | < 0.001 | < 0.001 | < 0.001 | < 0.001 |
| Reproductive efficiency | < 0.001 | 0.297 | < 0.001 | < 0.001 |
